# Esclerose Tuberosa: Achados Incomuns em Contexto de uma Doença Rara

**DOI:** 10.36660/abc.20220147

**Published:** 2023-01-31

**Authors:** Ines Oliveira, Rafaela Lopes, Isabel Cruz, Bruno Bragança, João Azevedo, Aurora Andrade

**Affiliations:** Centro Hospitalar Tâmega e Sousa EPE Penafiel Portugal Centro Hospitalar Tâmega e Sousa,1 EPE, Penafiel – Portugal

**Keywords:** Esclerose Tuberosa/complicações, Diagnóstico por Imagem, Neoplasias, Tumor Supressor/genética, Aterosclerose, Lipomatose Múltipla Familiar

## Descrição do caso

Mulher de 40 anos de idade, diagnosticada com esclerose tuberosa (ET) com um ano de idade, com déficit cognitivo, epilepsia e angiomiolipoma renal, encaminhada à consulta de cardiologia com queixas de cansaço. A paciente não tinha história familiar de ET. Embora sua mãe não conseguisse confirmar se a paciente tinha sido submetida a um teste genético, o diagnóstico de ET foi feito de acordo com os critérios revisados do *International Tuberous Sclerosis Consensus Group* , pela presença de vários critérios *majores* , tais como máculas hipomelanóticas, angiofribromas da face, displasia cortical e nódulos subependimários.

**Figura 1:**
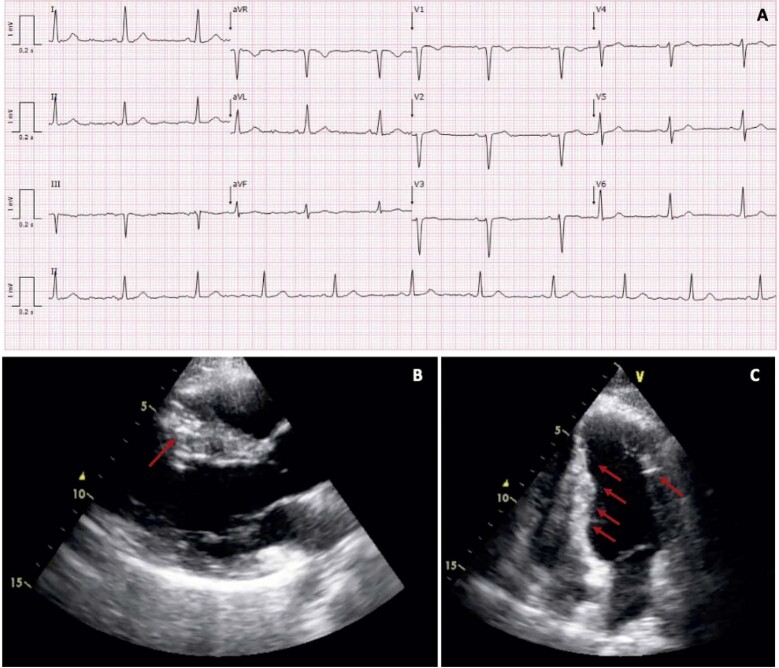
– A) Eletrocardiograma mostrando ritmo sinusal, má progressão da onda R nas derivações pré-cordiais anteriores e anormalidades inespecíficas de repolarização: B,C) PSLAX e ecocardiograma transtorácico quatro câmaras mostrando lesões hiperecogênicas no miocárdio localizadas na parede anterosseptal, inferosseptal e anterolateral do ventrículo esquerdo (➞)

**Figura 2:**
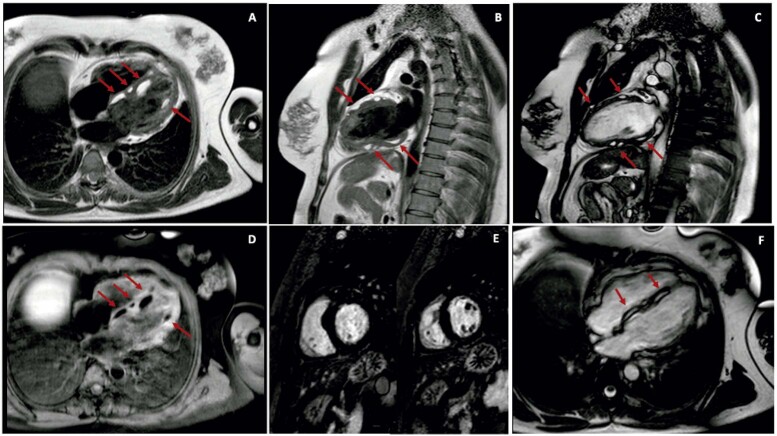
– A,B) Imagens ponderadas em T1 mostrando múltiplas lesões no miocárdio com sinal hiperintenso e homogêneo. Não há sinais de outras massas intracardíacas; C) imagens ponderadas em T2 mostrando lesões intramiocárdicas com hipersinal homogêneo. Não há sinais de outras massas intracardíacas; D) Sequência de cine ressonância mostrando supressão síncrona do sinal com a aplicação de pulsos de saturação, obtendo-se intensidade de sinal similar ao tecido adiposo adjacente; E) Ausência de realce tardio com gadolínio; F) Presença de chemical shift, destacando a interface entre as lesões no miocárdio e o miocárdio, revelando a natureza lipídica das lesões.

Na avaliação cardiovascular, a paciente não apresentava dispneia de esforço, dor torácica, palpitações ou história de síncope. No exame físico, observavam-se múltiplos angiofibromas cutâneos e máculas hipomelanóticas, auscultação cardíaca sem sopros cardíacos ou sinais de congestão. O eletrocardiograma (ECG) mostrou alteração inespecífica da repolarização ( Figura 1 ), e nenhum evento disrítmico foi detectado pelo Holter. O ecocardiograma transtorácico revelou múltiplas lesões hiperecogênicas intracardíacas, sem obstrução de fluxo sanguíneo ou disfunção valvar, e função sistólica ventricular preservada ( Figuras 1B e C ). A ressonância magnética cardíaca (RMC) identificou a natureza das lesões ( Figuras 2C-H ) – sinal hiperintenso e homogêneo nas imagens ponderadas em T1 e em T2 (A, B, C), com supressão uniforme de gordura com a aplicação de pulsos de saturação, com intensidade similar ao tecido adiposo (D). As lesões também mostraram alteração química, revelando sua natureza lipomatosa (F). Sem evidência de perfusão, realce tardio pelo gadolínio (E) ou outras massas. A paciente permaneceu assintomática após cinco dias de seguimento, realizando ECG e ecocardiogramas anuais, sem aumento no número ou tamanho dos lipomas.

## Discussão

A ET é uma doença dominante autossômica caracterizada pelo crescimento de neoplasmas benignos em múltiplos órgãos.^1 , 2^ A doença pode ser familiar ou se desenvolver como um caso esporádico.^2^ O cérebro e a pele são os órgãos mais afetados, com o desenvolvimento de tumores cerebrais, nódulos subependimários e displasia cortical, máculas hipomelanóticas e rabdomiomas cardíacos, cistos renais e angiomiolipomas também caracterizam a doença.^1 , 2^

Rabdomiomas são a manifestação cardíaca clássica, sendo um dos critérios diagnósticos;^1^ são geralmente assintomáticos, com regressão espontânea durante a infância, embora às vezes, sintomas possam se desenvolver devido ao tamanho e local do tumor, levando à obstrução valvar e sintomas de insuficiência cardíaca.^1 , 2^

No nosso caso, a paciente foi à consulta com cardiologista quando tinha 40 anos de idade. A avaliação cardíaca revelou múltiplos lipomas cardíacos – uma característica que, embora por vezes descritos na ET, têm importância desconhecida e não têm papel no diagnóstico.^3^ No entanto, a difusão de técnicas de imagem multimodal levou a um crescente reconhecimento da presença de lipomas cardíacos em pacientes com ET, levantando a questão sobre o rastreio de outras características da doença em pacientes com lipomas cardíacos identificados incidentalmente. Os lipomas podem ser resultado natural da regressão do rabdomioma ou, em casos de pacientes com ET e angiomiolipomas renais, podem representar focos metastáticos de lesões renais.^3 , 4^ Esse último, contudo, requereria confirmação por biópsia, o que não foi realizado nos casos publicados.^3 - 5^

A gordura, mais comumente encontrada ao redor do músculo cardíaco; está fortemente relacionada com fatores de riscos como aterosclerose e resistência insulínica, e portanto com prognóstico cardiovascular adverso.^6^ Embora o depósito de gordura no miocárdio possa ocorrer como um consequência do envelhecimento, ela é principalmente encontrada em condições clínicas em que os miócitos são substituídos por tecido fibroadiposo devido a dano prévio irreversível no miocárdio (necrose, infecção) ou no contexto da cardiomiopatia arritmogênica.^7^ No entanto, apesar da quantidade variável de adipócitos nessas condições, o desenvolvimento de lipomas cardíacos não é comum.^8^ Esses consistem em tumores benignos encapsulados por tecido fibroso, cuja patogênese não é completamente esclarecida.^7 , 8^ Geralmente são lesões silenciosas, embora há relatos de sintomas devido à interferência com outras estruturas cardíacas.^8^

Exames de imagens têm um papel essencial no diagnóstico de massas intracardíacas.^8 , 9^ Na tomografia computadorizada, os lipomas cardíacos são lesões bem circunscritas que apresentam um sinal homogêneo de baixa atenuação.^5 , 8^ Na RMC, essas lesões apresentam mesma intensidade de sinal do tecido adiposo torácico nas imagens ponderadas em T1 e T2;^7 - 9^ apresentam um hipersinal nas imagens em T1, as quais são fortemente suprimidas com a aplicação de pulsos adicionais de saturação de gordura.^8 , 9^ Os lipomas cardíacos também apresentam um artefato de *chemical shift* , representado por uma linha preta sobre a interface lipídica-aquosa, sem perfusão ou realce tardio com gadolínio.^9^

Além do diagnóstico, os exames de imagem são cruciais no seguimento para avaliar o crescimento da massa e possível interferência com estruturas adjacentes. A ecocardiografia tem um papel bem estabelecido na avaliação da função ventricular e valvular durante o seguimento do paciente.^1^ A RMC possibilita melhor caracterização do tecido, sem radiação ionizante, e deve ser utilizada quando há suspeita de crescimento ou interferência mecânica.^1 , 7 , 9^

Embora a maioria dos lipomas não cresçam e permaneçam assintomáticos ao longo do tempo, é importante investigar sinais e sintomas de insuficiência cardíaca, o que requereria terapia específica, uma vez que a maioria desses pacientes apresentam déficit cognitivo, o que impõe maior desafio ao reconhecimento de sintomas e intervenção.^1 , 2 , 7^

O objetivo deste relato caso é destacar características específicas de uma doença rara, bem como o papel dos exames de imagem no diagnóstico, seguimento e manejo apropriado do envolvimento cardíaco.
