# Supplementary material for: Esclerose Tuberosa: Achados Incomuns em Contexto de uma Doença Rara
Source: Arq Bras Cardiol. 2023 Jan 31;120(1):e20220147. [Article in Portuguese] doi: 10.36660/abc.20220147 (PMC10389102; doi:10.36660/abc.20220147)
Supplement: Translation

# Translation: Tuberous Sclerosis: Unusual Findings in the Setting of a Rare Disease

**Authors:** Ines Oliveira, Rafaela Lopes, Isabel Cruz, Bruno Bragança, João Azevedo, Aurora Andrade

**Affiliations:** 1 Portugal Centro Hospitalar Tâmega e Sousa, EPE, Penafiel – Portugal

**Keywords:** Tuberous Sclerosis/complications, Diagnostic Imaging, Neoplasms, Tumor Supressor/genetics, Atherosclerosis, Familial Multiple Lipomatosis

## Case Description

A 40-year-old woman diagnosed with tuberous sclerosis (TS) at the age of one, with cognitive impairment, epilepsy and renal angiomyolipomas, was referred to Cardiology for complaints of fatigue. She had no known family history of TS. Although her mother could not confirm if a genetic test had been performed, the patient had a definite diagnosis of TS, according to International Tuberous Sclerosis Consensus Group revised criteria, due to the presence of several *major* criteria, as hypomelanotic macules, facial angiofibromas, cortical dysplasia and subependymal nodules.

On cardiovascular evaluation, she had no dyspnea on exertion, chest pain, palpitations or history of syncope. On physical examination, she had multiple cutaneous angiofibromas and hypomelanotic macules, cardiac auscultation with no murmurs or signs of congestion. Electrocardiogram (ECG) showed nonspecific repolarization abnormalities ( Figure 1A ) and no arrhythmic events were detected by the Holter method. Transthoracic echocardiography revealed the presence of multiple intracardiac hyperechogenic lesions, with no blood flow obstruction or valvular impairment, and preserved ventricular systolic function ( Figures 1B and C ). Cardiac magnetic resonance (CMR) showed the nature of the lesions ( Figures 2C-H ) – homogeneous high signal intensity in T1 and T2-weighted images (A, B, C), with uniform suppression with the application of fat-saturation pulses, with an intensity similar to adipose tissue (D). The lesions also showed *chemical shift* , revealing its lipomatous nature (F). No evidence of perfusion, late gadolinium enhancement (E) or other masses. The patient remained asymptomatic after five years of follow-up, performing an annual ECG and echocardiogram, with no increase in the number or size of lipomas.

**Figure 1:** – A) Electrocardiogram depicting sinus rhythm, poor R wave progression in anterior precordial leads and nonspecific repolarization abnormalities; B,C) PSLAX and four-chamber transthoracic echocardiogram showing hyperechogenic intramyocardial lesions located on the anteroseptal, inferoseptal and anterolateral wall of the left ventricle (➞)

**Figure 2:** – A,B) T1-weighted images depicting multiple intramyocardial lesions with homogeneous high signal intensity; C) T2-weighted images showing intramyocardial lesions with homogeneous high signal intensity. No signs of other intracardiac masses; D) Cine sequence showing synchronous signal suppression with the application fat-saturation prepulses, acquiring a signal intensity similar to the adjacent adipose tissue; E) Absence of late gadolinium enhancement; F) Presence of chemical shift, highlighting the interface between the intramyocardial lesions and the myocardium, revealing its adipose nature.

## Discussion

TS is an autosomal dominant disorder characterized by multiorgan growth of benign neoplasms.^1 , 2^It may be familiar or develop as a sporadic case.^2^Brain and skin are the mostly affected organs, with the development of brain tumors, subependymal nodules and cortical dysplasia, hypomelanotic macules and cutaneous angiofibromas. The presence of hamartomas, cardiac rhabdomyomas, renal cysts and angiomyolipomas also characterize the disease.^1 , 2^

Rhabdomyomas are the classic cardiac manifestation, being one of the *major* diagnostic criteria.^1^They are usually asymptomatic and show spontaneous regression during childhood, though sometimes symptoms may develop due to tumor size and location, leading to valvular obstruction and heart failure symptoms.^1 , 2^

In our case, the patient attended a Cardiology appointment when she was 40 years old. Cardiac evaluation revealed multiple cardiac lipomas – a feature that although sometimes described in TS, has unknown significance and no role in the diagnosis.^3^However, the spreading of multimodality imaging has led to an increasing recognition of the presence of cardiac lipomas in TS patients, raising the issue of screening other features of the disease in patients with incidentally found cardiac lipomas. They might be the natural result of rhabdomyoma regression or, in cases of TS patients with renal angiomyolipomas, might represent metastatic foci of the renal lesions.^3 , 4^However, the latter would require confirmation by biopsy, which has not been performed in the published cases.^3 - 5^

Fat is more commonly found surrounding the heart muscle; it is strongly related to cardiovascular risk factors such as atherosclerosis and insulin resistance, and hence with adverse cardiovascular prognosis.^6^Although intramyocardial fat deposition may occur as a consequence of ageing, it is mainly found in clinical conditions where myocytes are replaced by fibro-adipose tissue due to previous irreversible myocardial damage (necrosis, infection) or in the setting of arrhythmogenic cardiomyopathy.^7^However, despite variable amounts of adipocytes in these conditions, the development of cardiac lipomas is uncommon.^8^These are benign tumors encapsulated by fibrous tissue, whose pathogenesis is not completely understood.^7 , 8^They are usually silent lesions, although symptoms have been reported due to interference with other cardiac structures.^8^

Imaging plays an essential role in the diagnosis of intracardiac masses.^8 , 9^On computed tomography scan, cardiac lipomas are well-circumscribed lesions that display a homogenous low-attenuation signal.^5 , 8^On CMR, these lesions have the same signal intensity of chest adipose tissue on T1 and T2-weighed images;^7 - 9^they display a high-signal intensity on T1-weighed images that is markedly suppressed with additional fat-saturation pulses.^8 , 9^Cardiac lipomas also depict a chemical-shift artefact, represented by the black boundary artefact on the fat-water interface, with no perfusion or late gadolinium enhancement.^9^

Besides diagnosis, imaging is crucial in follow-up, in order to evaluate mass growing and possible interference with adjacent structures. Echocardiography has a stablished role in follow-up, for assessment of ventricular and valvular function.^1^CMR provides better tissue characterization without ionizing radiation and should be used when growth or mechanical interference is suspected.^1 , 7 , 9^

Although most cardiac lipomas do not grow and remain asymptomatic over time, it is important to search for heart failure symptoms and signs, which would require specific therapy, as most of these patients have cognitive impairment which poses an additional challenge to symptom recognition and intervention.^1 , 2 , 7^

The purpose of this case report is to highlight specific features of a rare disease and the role of imaging in diagnosis, follow-up and appropriate management of cardiac involvement.
